# Predictive value of maximum tumor dissemination (Dmax) in lymphoma patients treated with CD19-specific CAR T-Cells

**DOI:** 10.1186/s40644-025-00959-w

**Published:** 2025-11-22

**Authors:** Michael Winkelmann, Philipp Achhammer, Viktoria Blumenberg, Kai Rejeski, Veit L. Bücklein, Christian Schmidt, Gabriel T. Sheikh, Matthias Brendel, Jens Ricke, Michael von Bergwelt-Baildon, Marion Subklewe, Wolfgang G. Kunz

**Affiliations:** 1 Department of Radiology, University Hospital, LMU Munich, Marchioninistr. 15, 81377 Munich, Germany; 2 Laboratory for Translational Cancer Immunology, Gene Center of the LMU Munich, Munich, Germany; 3 German Cancer Consortium (DKTK) and Bavarian Center for Cancer Research (BZKF), Partner Site Munich, Munich, Germany; 4 Department of Medicine III, University Hospital, LMU Munich, Munich, Germany; 5 Cellular Immunotherapy Program, Massachusetts General Hospital Cancer Center, Harvard Medical School, Charlestown and Broad Institute of Harvard University and Massachusetts Institute of Technology, Cambridge, MA USA; 6 Department of Nuclear Medicine, University Hospital, LMU Munich, Munich, Germany; 7 Comprehensive Cancer Center München-LMU (CCCM LMU), LMU Munich, Munich, Germany; 8 DZNE – German Center for Neurodegenerative Diseases, Munich, Germany; 9 Munich Cluster for Systems Neurology (SyNergy), University of Munich, Munich, Germany

**Keywords:** Lymphoma, CAR t-cell therapy, ^18^F-FDG PET/CT, Dissemination features, Dmax, Dmax_bulk_, Ann-Arbor, Deauville-Score, Lugano criteria

## Abstract

**Objectives:**

CD19-specific chimeric antigen receptor T-cell therapy (CART) has emerged as effective treatment for relapsed or refractory (r/r) lymphoma. The maximum distance (Dmax) of lymphoma lesions holds potential as prognostic imaging biomarker in lymphoma treated with conventional therapies, but evidence in the context of CART remains scarce and further studies are needed to clarify its clinical relevance. We evaluated Dmax at baseline imaging as a potential prognostic tool for assessment of metabolic and overall response, progression-free survival (PFS) and overall survival (OS).

**Material & methods:**

Consecutive r/r lymphoma patients with (PET/)CT imaging at baseline (BL) before lymphodepletion and subsequent CAR T-cell transfusion were included. Dmax was measured in cm at BL. Patients were divided by tertiles into three equal sized groups according to Dmax. Ann Arbor stages were calculated at baseline and the sum of product diameters (SPD) was used to represent tumor burden (TB). Overall response according to Lugano criteria and the Deauville score were determined at day 90 PET/CT imaging.

**Results:**

Thirty-nine patients met the inclusion criteria. Median Dmax was 40.0 cm (IQR: 16.4–70.3 cm) at BL. Median TB decreased from BL with 4,095 mm^2^ to 770 mm^2^ at FU imaging. Median TB at BL was significantly higher in the Dmax intermediate and high group compared to the Dmax low group (*p* = 0.005) with 7,222 mm^2^ (IQR: 3,355–11,941 mm^2^), 4,649 mm^2^ (IQR: 2,376–10,406 mm^2^) and 1,739 mm^2^ (IQR: 715–7,402 mm^2^), respectively. Dmax intermediate and high group showed significantly higher Ann Arbor stages (*p* < 0.001). The survival analysis revealed a significantly (*p* = 0.030) shorter PFS in the Dmax high group compared to the other patients (91 vs. 364 days), but no relevant differences in OS (*p* = 0.151).

**Conclusions:**

Patients with high Dmax showed a shorter PFS, but no significant differences in OS. Dmax is a useful parameter for characterizing tumor dissemination, which could also be incorporated into scores due to its interval scale.

**Supplementary Information:**

The online version contains supplementary material available at 10.1186/s40644-025-00959-w.

## Background

CD19 specific chimeric antigen receptor (CAR) T-cell therapy with patient-derived T-cells is now established as novel and effective treatment for relapsed or refractory (r/r) non-Hodgkin lymphoma (NHL) [1] and demonstrated efficacy in r/r large B-cell lymphoma (LBCL) [2–4], follicular lymphoma (FL) [3, 4], and mantle-cell lymphoma (MCL) [5]. CART has significantly improved progression-free survival (PFS) and overall survival (OS) [6]. 

Imaging in NHL is routinely performed using positron emission tomography-computed tomography (PET-CT) with 18 F-fluorodeoxyglucose (18 F-FDG) or computed tomography (CT) to quantify tumor burden, lymphoma dissemination and evaluate extranodal disease [7]. 18 F-FDG PET/CT is prognostic in several lymphoma subtypes [8]. In clinical practice, the Ann Arbor classification, which is used for both Hodgkin’s lymphoma and NHL, is most commonly applied for the assessment of dissemination. It represents the equivalent to the TNM staging for solid tumors and is subdivided into four stages (AAS I-IV). The staging is based on both the location of the lymphoma tissue and the systemic symptoms caused by the lymphoma (“B-symptoms”: night sweats, weight loss or fever) [9–11]. 

In current trials, the most widely adopted response assessment of NHL is based on the Lugano criteria from 2014 that can also be applied in context of CART [7, 12–14]. Evaluation is based on morphologic criteria like change in sum of product diameter (SPD) and metabolic response, which is evaluated using the 5-point Deauville scale (DS) [12, 15]. Other morphologic and metabolic imaging biomarkers are becoming increasingly important in risk assessment of NHL treated with CART and numerous studies indicate the additional prognostic value of imaging parameters [16]. In addition, new scores such as the International Metabolic Prognostic Index (IMPI), which includes the metabolic tumor volume, have been found to have predictive power in the context of CART [8, 17]. 

The maximum distance (Dmax) of lymphoma lesions, which represents the greatest span between lesions on imaging, has shown potential as a prognostic imaging biomarker in lymphoma treated with conventional therapies, but evidence in the context of CART remains scarce and further studies are needed to clarify its clinical relevance [18–20]. We evaluated Dmax at baseline imaging as a prognostic tool for assessment of metabolic and overall response, progression-free survival (PFS) and overall survival (OS).

## Materials and methods

### Study design and population

All medical records and imaging studies underwent review with approval from the Ludwig Maximilian University (LMU) Munich Institutional Review Board (LMU Ethics Committee, project number 19–817). In addition, all individual patients in the study gave their informed consent. The study population was based on a registry of all consecutive patients who were treated with standard-of-care CD19 specific CART products at the Comprehensive Cancer Centre Munich of the Ludwig-Maximilian University Munich (CCCM^LMU^) between January 2019 and March 2023 (data cutoff) and was analyzed retrospectively. The following inclusion criteria were applied:

1. Patients with r/r lymphoma (LBCL and MCL).
2. Available CT or PET-CT imaging studies at baseline (≤ 2 weeks before CART).
3. Any measurable disease by imaging according to Lugano criteria [12].

The following exclusion criteria were applied:

1. No measurable disease on imaging according to Lugano criteria [12].
2. Any non-diagnostic or incomplete imaging studies.

Histologic diagnoses were evaluated by expert pathologists. Patients underwent lymphodepletion with fludarabine and cyclophosphamide according to the manufacturers’ instructions. Bridging therapy was defined as systemic therapy or local radiation between time of indication and lymphodepletion. The international prognostic index (IPI) was calculated using age, Eastern Co-operative Oncology Group (ECOG) performance status, Ann Arbor stage, serum lactate dehydrogenase (LDH), and extranodal involvement [21]. 

### Acquisition of 18 F-FDG PET-CT and CT imaging

PET/CT images were acquired approximately 60 min after tracer injection (159–275 MBq weight-adapted) and for the FDG PET/CT contrast-enhanced or unenhanced CTs using a slice thickness of 3 mm 100 kVp, 100–400 mAs, and dose modulations were performed for attenuation correction. The following scanners were used: Biograph 64 and Biograph mCT (Siemens Healthineers, Germany) or Discovery 690 (GE Healthcare, USA). The following reconstruction algorithms were used: Bio-graph 64, TrueX (3 iterations, 21 subsets) with Gaussian post-reconstruction smoothing (2 mm full width at half-maximum); Biograph mCT, TrueX (3 iterations, 21 subsets); Discovery 690, VUE Point FX algorithm with 2 iterations and 36 subsets. All systems resulted in a PET image with a voxel size of 2 × 2 × 2 mm^3^.

### Imaging and response assessment

**Fig. 1:**
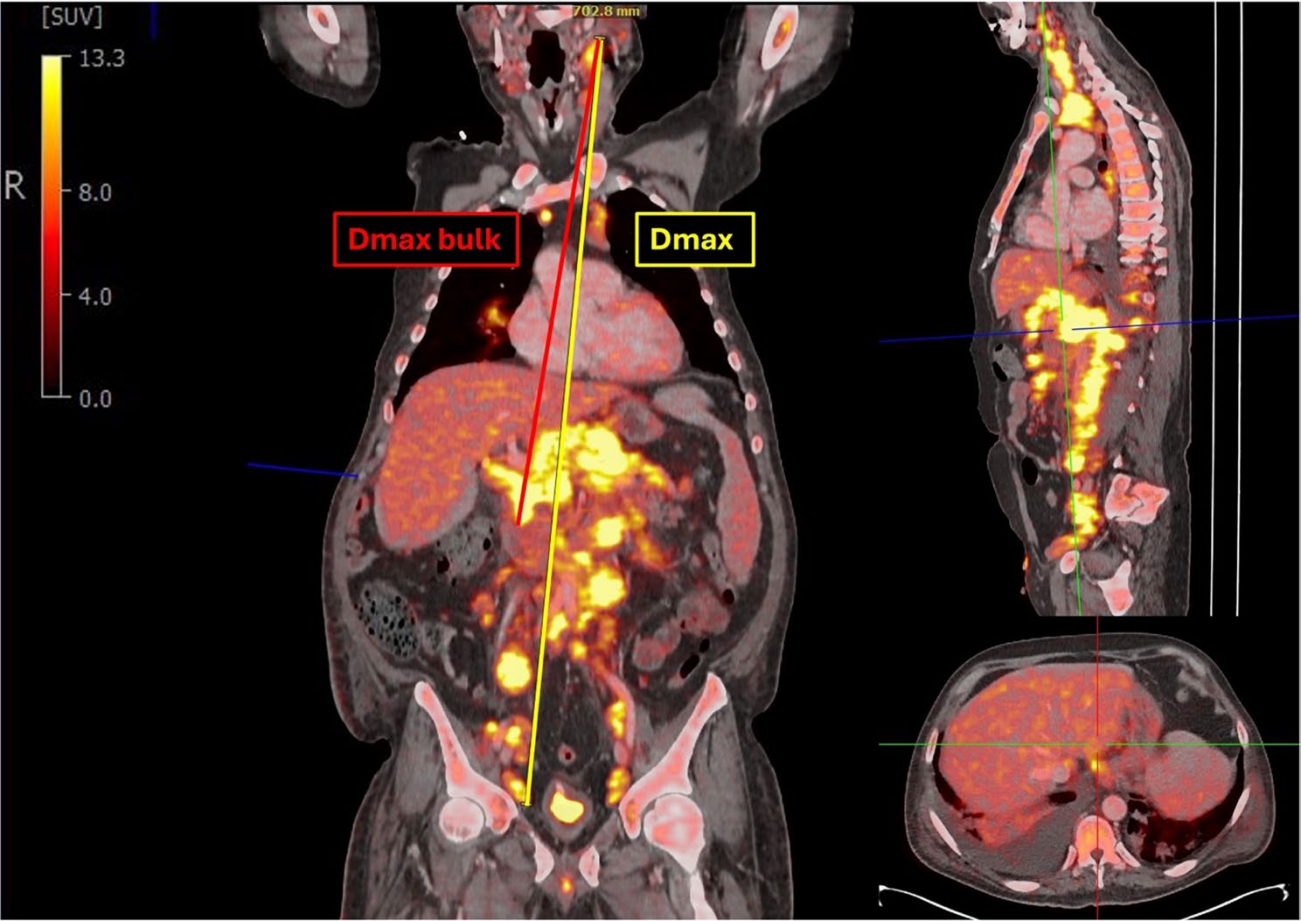
Measurement of Dmax and Dmax bulk. Illustration of measurement procedure of Dmax (yellow) and Dmax bulk (red) in multiplanar reconstruction. Dmax was defined as the distance of the most distant lymphoma manifestations with end-to-end measurement. For Dmaxbulk, the measurement was performed from the largest lymphoma manifestation to the lesion furthest away from it

Dissemination features Dmax and Dmax_bulk_ were measured at baseline (BL) CT or PET/CT imaging using multiplanar reconstruction (MPR) in Visage Imaging PACS software (Visage Imaging GmbH; Berlin, Germany) as shown in Fig. 1. Dmax was defined as the distance of the most distant lymphoma manifestations with end-to-end measurement. For Dmax_bulk_, the measurement was performed from the largest lymphoma manifestation to the lesion furthest away from it.

Overall response was determined based on Lugano criteria [12]. The sum of product diameters (SPD) of up to six target lesions according to Lugano criteria was used to represent tumor burden (TB). Spleen size was measured in craniocaudal extension with splenomegaly being defined by a vertical length >13.0 cm. Depth of response (DoR) was determined as the percentage decrease or increase of SPD from baseline (BL) to follow-up (FU) imaging at day 90. Overall metabolic activity was determined in 18 F-FDG PET/CT using the 5-point Deauville score (DS).

All imaging analyses for structured Lugano assessment were performed at the timepoints of BL and if available FU with dedicated trial reporting software mintLesion 3.8 (mint Medical GmbH; Heidelberg, Germany). The reading and measurements were approved by consensus reading of two board certified radiologists with at least 7 years of experience in radiology and nuclear medicine.

### Survival analysis

Progression-free survival (PFS) was defined as the time from initiation of CART treatment to progression of lymphoma based on imaging, unequivocal clinical findings, and/or histologic confirmation. Overall survival (OS) was defined as the time from the start of therapy to death from any cause. For survival analysis, PFS and OS were visualized using Kaplan-Meier survival curves.

### Statistical analysis

Spearman’s correlation and linear regression studied association of Dmax and Dmax_bulk_ with SPD, and Dmax and Dmax_bulk_ with AAS. Optimal cut-off values for Dmax and Dmax_bulk_ were determined using the MaxStat test. Survival functions were obtained with Kaplan-Meier estimates. Log-rank (Mantel-Cox) tests were performed to compare survival curves and calculate hazard ratios. Statistical significance between groups was explored by non-parametric Mann-Whitney test and Kruskal-Wallis for absolute values. Fisher’s exact and Chi-square test were used for comparison of percentages. All statistical analyses were performed using GraphPad Prism 10. P-values below 0.05 were considered to indicate statistical significance.

## Results

### Patient characteristics

**Fig. 2:**
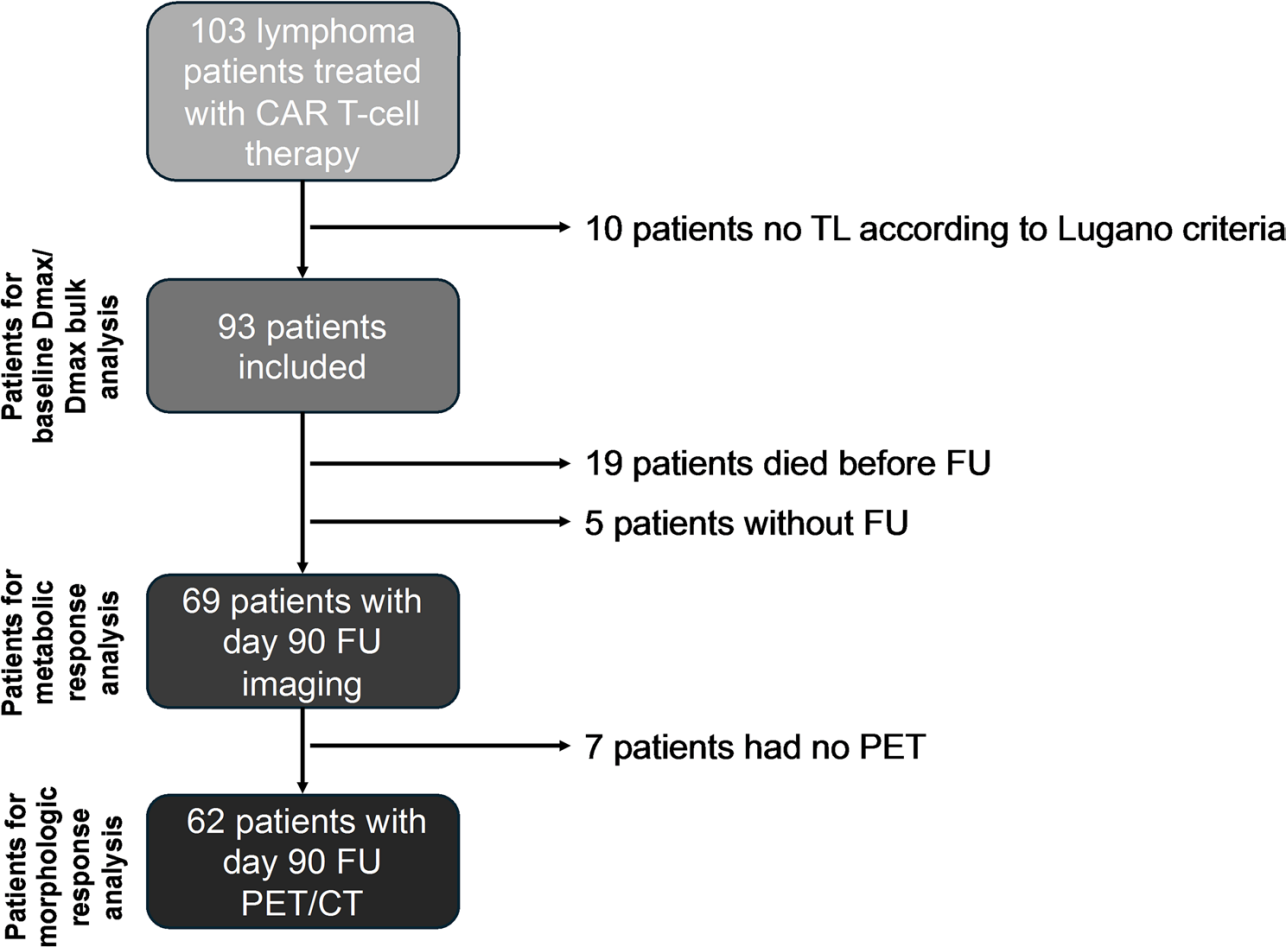
Flow chart. Of the 103 patients treated with CART, 10 patients were excluded due to missing target lesions according to Lugano criteria. 93 patients could be included for the analysis of the baseline data of Dmax and Dmax bulk. For the response analysis, 5 patients had no FU imaging and another 19 patients died before the day 90. Sixty-nine patients were analyzed in the morphological response analysis and 62 patients in the metabolic response analysis, as 7 patients had only a CT but no PET at FU

Ninety-three out of 103 patients met the inclusion criteria for the Dmax and Dmax bulk analysis at baseline. Ten patients had to be excluded because they had no measurable target lesion (TL) according to Lugano criteria. For the response analysis, 5 patients had no FU imaging and another 19 patients died before the day 90. Sixty-nine patients were analyzed in the morphological response analysis and 62 patients in the metabolic response analysis, as 7 patients had only a CT but no PET at FU. A detailed flowchart is provided as Fig. 2.

Median patient age was 64 years and 35 patients (38%) were female. According to Ann Arbor staging system, 14 patients (15%) had stage I, 24 (26%) stage II, 14 (15%) stage III and 41 patients (44%) stage IV disease. Bridging therapy was given to 68 (73%) of the 93 patients. Median SPD at baseline was 4,095 mm2 with an IQR of 1,568–10,566 mm2. Median Dmax was 40 cm with an IQR of 16.4–70.3 cm and median Dmaxbulk 35.8 cm with an IQR of 15.25–51.7 cm. At day 90 FU 38 patients (55%) had complete response (CR), 9 (13%) partial response (PR), 2 (3%) stable disease (SD) and 20 patients (29%) had progressive disease (PD) according to Lugano criteria. For the analysis of differences in patient characteristics across Dmax-based groups, patients were stratified into three equal-sized groups based on tertiles: low, intermediate and high. The detailed results are shown in Table 1. The MaxStat test identified Dmax > 57.8 cm and Dmaxbulk > 55.2 cm as the optimal cut-offs for predicting PFS.

**Table 1:** Patient characteristics

		Total (*n* = 93)	Low (*n* = 31)	Intermediate (*n* = 31)	High (*n* = 31)
Age (median)		64	64	61	66
Gender / Sex	Female:	35 (38%)	16 (52%)	12 (39%)	7 (23%)
	Male:	58 (62%)	15 (48%)	19 (61%)	24 (77%)
Lymphoma Entity	LBCL:	80 (86%)	26 (84%)	29 (94%)	25 (81%)
	MCL:	13 (14%)	5 (16%)	2 (6%)	6 (19%)
Ann Arbor Stage	I: II: III: IV:	14 (15%) 24 (26%) 14 (15%) 41 (44%)	12 (39%) 14 (45%) 2 (6%) 3 (10%)	1 (3%) 8 (26%) 4 (13%) 18 (6%)	1 (3%) 2 (6%) 8 (26%) 20 (65%)
IPI	0: 1: 2: 3: 4: 5:	4 (4%) 20 (22%) 22 (24%) 24 (26%) 16 (17%) 7 (7%)	4 (13%) 14 (45%) 8 (26%) 5 (16%) 0 (0%) 0 (0%)	0 (0%) 5 16%) 4 (13%) 13 (42%) 7 (23%) 2 (6%)	0 (0%) 1 (3%) 10 (32%) 6 (19%) 9 (29%) 5 (16%)
CAR T Product	Axicabtagene ciloleucel:	44 (47%)	17 (55%)	15 (48%)	12 (39%)
	Tisagenlecleucel:	31 (33%)	8 (26%)	11 (35%)	12 (39%)
	Brexucabtagene autoleucel:	13 (14%)	5 (16%)	2 (6%)	6 (19%)
	Lisocabtagene maraleucel:	5 (5%)	1 (3%)	3 (10%)	1 (3%)
Bridging	Yes - systemic - Local radiation No	68 (73%) - 61 (90%) - 7 (10%) 25 (27%)	17 (55%) - 16 (94%) - 1 (6%) 14 (45%)	27 (87%) - 25 (93%) - 2 (7%) 4 (13%)	24 (77%) - 20 (83%) - 4 (17%) 7 (23%)
SPD [mm^2^] (median)	Baseline FU2	4,095 769.9	1,739 346.5	7,222 1,164	4,649 484.5
Dmax [cm]	Median (IQR)	40.0 (16.4–70.3)	11.2 (0.0–16.4)	40.0 (38.4–53.0)	73.5 (70.2–78.5)
Dmax bulk [cm]	Median (IQR)	35.8 (15.3–51.7)	9.0 (0.0–16.0)	37.8 (32.3–47.2)	56.0 (49.5–63.5)
LDH prior Lymphodepletion [U/l]	Median (IQR)	279.0 (203.0–473.5)	229.0 (191.0–337.0)	359.0 (211.0–561.0)	301 (214.0–493.0)
Lugano Response FU	CR	38 (55%)	15 (60%)	12 (50%)	11 (55%)
	PR	9 (13%)	3 (12%)	3 (13%)	3 (15%)
	SD	2 (3%)	2 (8%)	0 (0%)	0 (0%)
	PD	20 (29%)	5 (20%)	9 (37%)	6 (30%)

CAR; chimeric antigen receptor; LBCL, large B cell lymphoma; IQR, interquartile range; MCL, mantle cell lymphoma; IPI, International Prognostic Index; SPD, sum of product of diameters; LDH, lactate dehydrogenase; FU1, follow-up 1 (day 30), FU2, follow-up 2 (day 90)

### Relation of Dmax and Dmax bulk groups with tumor burden and AAS

**Fig. 3:**
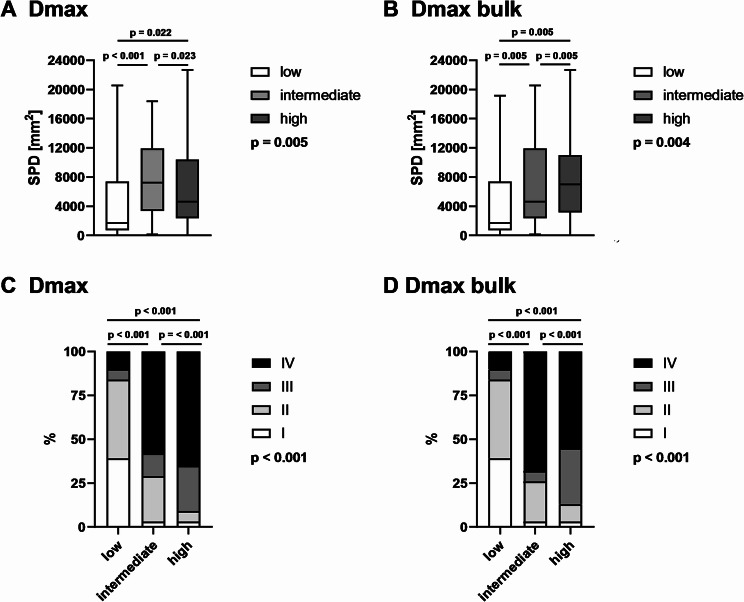
Relation of Dmax and Dmax bulk groups with tumor burden and Ann Arbor stage (AAS). The upper section shows box plots for the distribution of tumor burden measured as sum of product diameters (SPD) according to Lugano criteria. A division is made into 3 equal groups based on Dmax (**A**) and Dmax bulk (**B**). The lines within the boxes show the median. The bottom row shows the distribution of the AAS for the same groups divided according to Dmax (**C**) and Dmax bulk (**D**)

In a first sub-analysis we compared distribution of baseline TB between the three Dmax-based groups (Fig. 3A). Dmax low patients had a significant lower median SPD with 1,739 mm^2^ (IQR: 715–7,402 mm^2^), compared to intermediate (*p* < 0.001) with 7,222 mm^2^ (IQR: 3,355–11,941 mm^2^) and high patients (*p* = 0.023) with 4,649 mm^2^ (IQR: 2,376–10,406 mm^2^). Interestingly, the Dmax high group had a slightly lower SPD compared to the intermediate group (*p* = 0.022). In a similar way, when patients were subdivided according to their Dmax bulk (Fig. 3B), a significantly higher SPD was found with increasing Dmax group (*p* = 0.005). Correlation analysis showed a weak positive correlation between SPD and Dmax (*r* = 0.294) and Dmax bulk (*r* = 0.347), respectively.

Subsequently, we analyzed the relationship between Dmax and Dmax bulk groups and their distributional differences in Ann Arbor Stage (AAS). The higher the Dmax group, the higher the AAS tended to be (Fig. 3C; *p* < 0.001). Among the Dmax bulk groups, the low group had the lowest AAS (*p* < 0.001), while the intermedium group had a moderately higher AAS than the high group (Fig. 3D).

### Association of Dmax and Dmax bulk with depth of response (DoR)

**Fig. 4:**
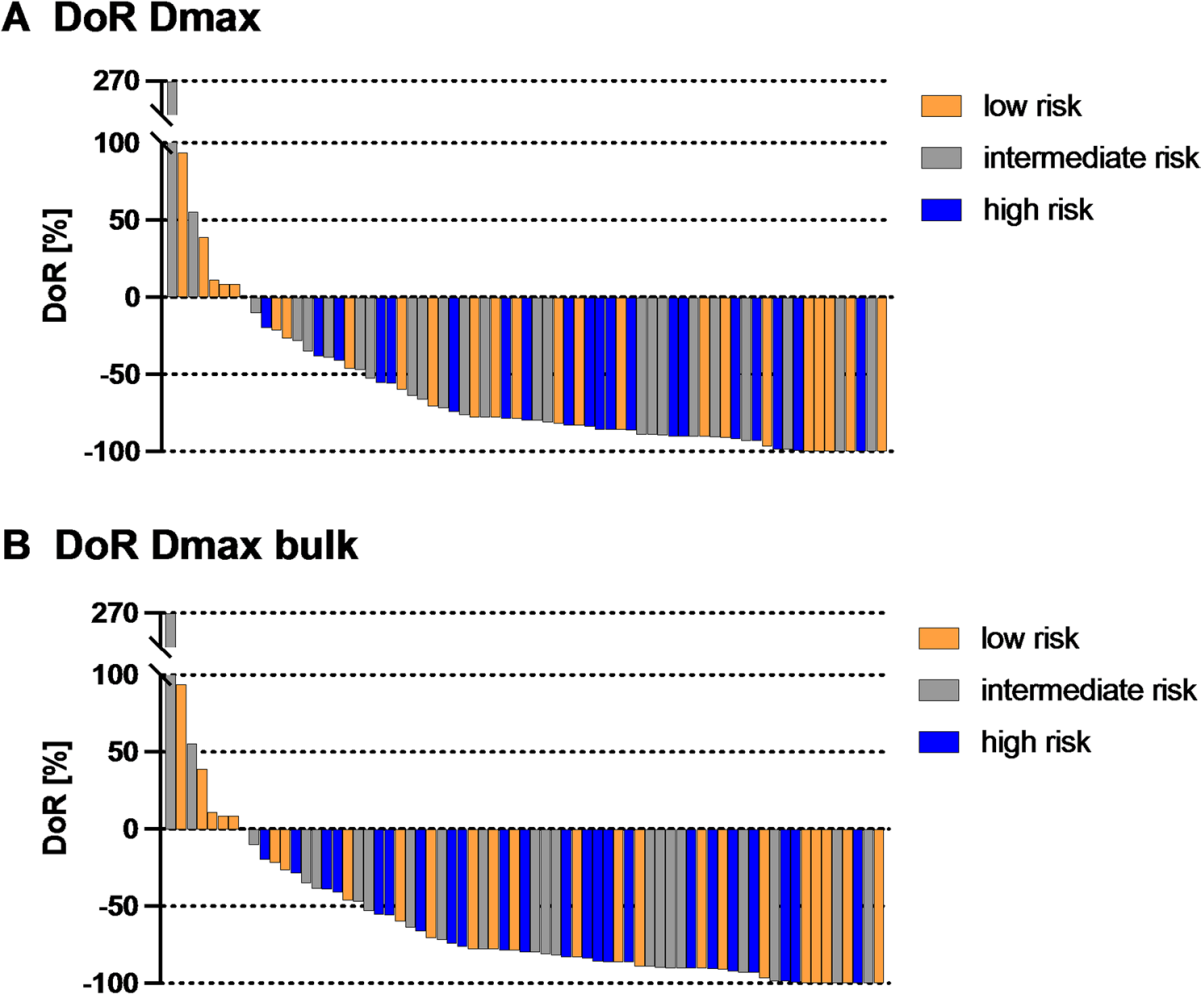
Association of Dmax and Dmax bulk with depth of response (DoR). Waterfall plot for the 69 patients with day 90 FU imaging according to the groups by Dmax (**A**) and Dmax bulk (**B**). Low patients are marked in orange, intermediate patients in gray and high patients in blue. The bars show the percentage change in sum of product diameters (SPD) from baseline to FU2 at 90 days. Negative values indicate a decrease and positive values an increase in tumor burden

In a next step, we analyzed the relationship of Dmax (Fig. 4A) and Dmax bulk (Fig. 4B) with depth of response (DoR) measured as change in SPD between baseline and 90-day FU2. The DoR calculated for all 69 patients with FU imaging and is illustrated as a waterfall plot in Fig. 4.

TB decreased in the median from BL with 4,095 mm^2^ to 770 mm^2^ at day 90 FU. In total, only 7 patients showed a positive DoR equivalent to an increase in TB. These seven patients belonged to either the low (five patients) or intermediate (two patients) group. All other patients had a negative DoR equal to a reduction of tumor burden an thus response to CART. There was no significant association of the different Dmax groups and DoR. An additional logistic regression analysis for response according to the Lugano criteria also yielded no significant results for either Dmax or Dmax bulk.

### Overall and metabolic response evaluation

**Fig. 5:**
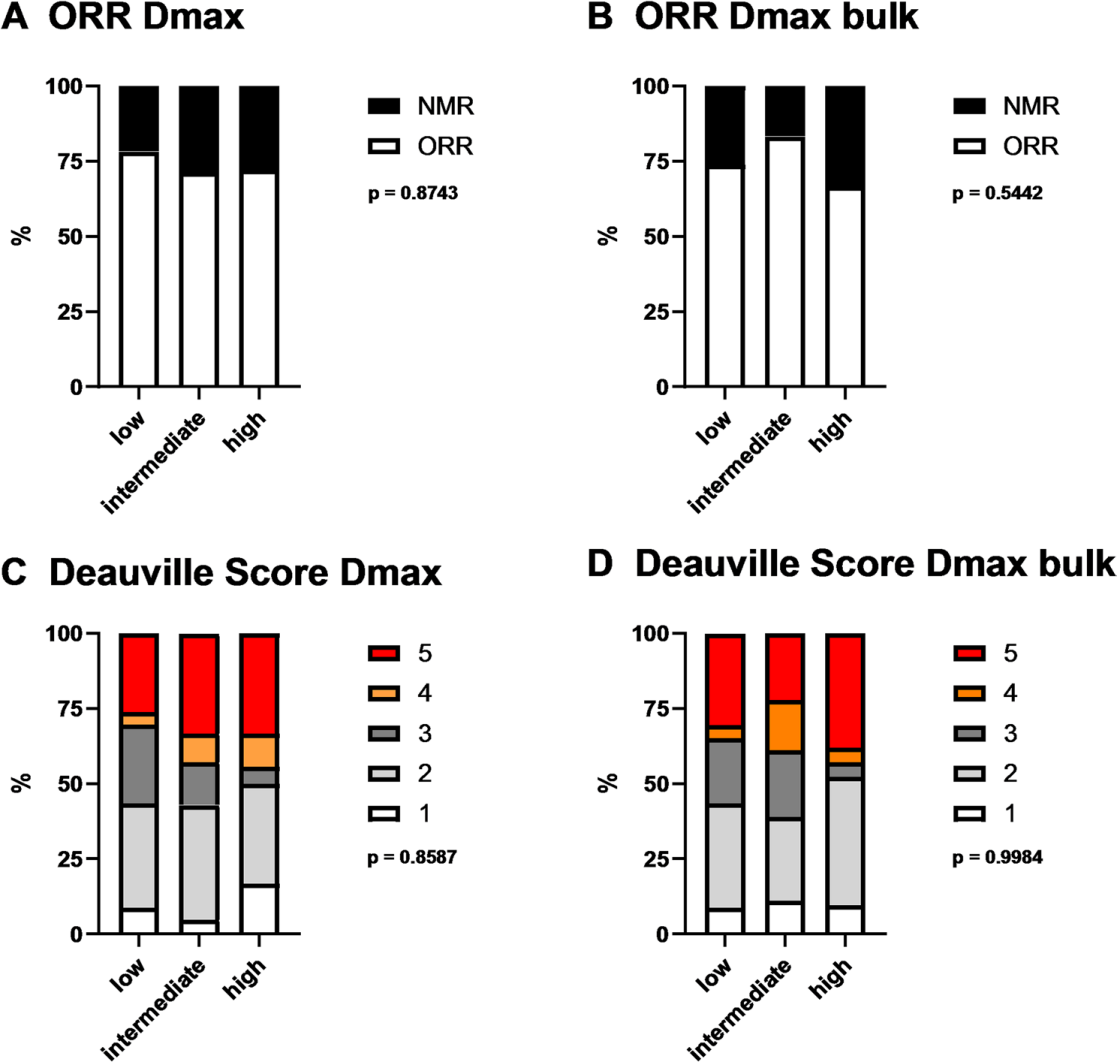
Evaluation of overall and metabolic response at 90-day follow-up. The upper half depicts the rate of ORR and NMR for the 3 groups according to Dmax (**A**) and Dmax bulk (**B**) at the time point of FU2 at 90 days. The lower part of the figure shows the Deauville score at FU2 for the same groups according to Dmax (**C**) and Dmax bulk (**D**)

In addition, to the analysis of SPD as morphological response, we analyzed overall and metabolic response at the day 90 FU. The overall response rates (ORR) for Dmax and Dmax bulk-based groups are displayed as stacked bar plots in Fig. 5A + B.

ORR was good in all groups with 78.3%, 71.4%, and 72.2% in Dmax-based low, intermediate, and high group, respectively. There were no significant differences for both the Dmax-based and Dmax bulk-based groups. There were minor differences in DS between Dmax intermediate and high patients (Fig. 5C) and Dmax bulk high patients had a slightly higher proportion of DS 5 (Fig. 5D) that were not statistically significant (both *p* > 0.05).

### Toxicity analysis

Toxicity analyses were additionally performed on a group-based level. Both Dmax and Dmaxbulk were significantly associated with treatment-related toxicity. Patients in the intermediate- and high-risk groups, defined by higher Dmax and Dmaxbulk values, had an increased risk of severe CRS (*p* = 0.014 and *p* = 0.007, respectively). Moreover, both parameters were associated with the occurrence of ICANS, with significant associations observed for Dmax (*p* = 0.014) and Dmaxbulk (*p* < 0.001). These findings are illustrated in Supplementary Fig. 1.

### Analysis of PFS and OS for Dmax and Dmax bulk-based groups

Finally, we assessed whether stratification by Dmax and Dmax bulk group is associated with PFS and OS. In univariate Cox regression (Supplementary Table 1), absolute Dmax and Dmaxbulk were not associated with PFS or OS. Using predefined cut-offs, Dmax > 57.8 cm predicted shorter PFS, while Dmaxbulk > 55.2 cm was linked to both shorter PFS and OS. ECOG, IPI, and elevated LDH were significant clinical risk factors, whereas Ann Arbor stage was prognostic only for PFS. SPD and continuous LDH showed no consistent effects, but elevated LDH remained prognostic for both endpoints. In multivariate analysis (Supplementary Table 2), combining imaging markers with clinical factors strengthened prognostic value, particularly Dmaxbulk > 55.2 cm with elevated LDH or ECOG, which remained significantly associated with poor PFS and OS.

**Fig. 6:**
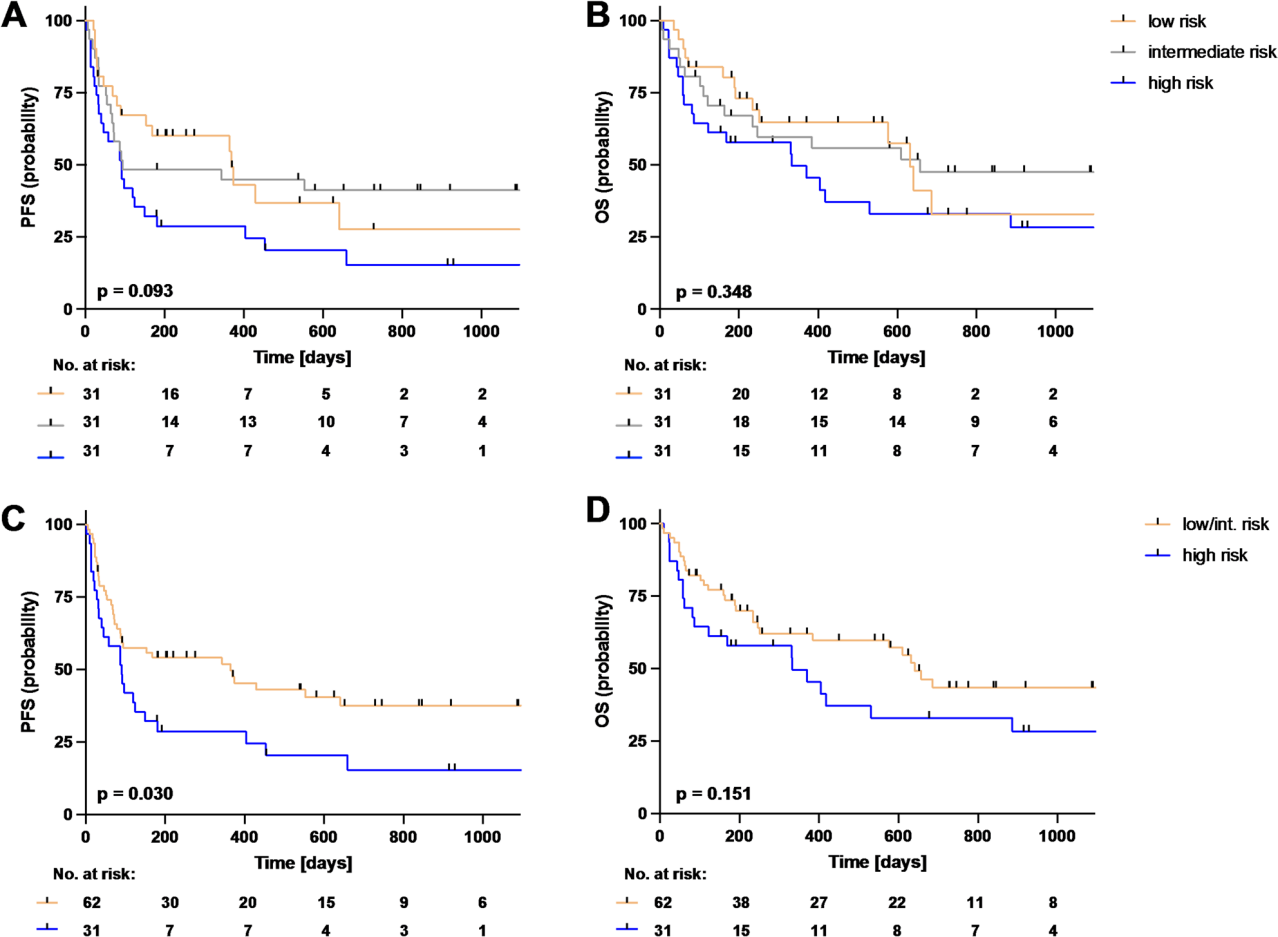
Survival analyses of progression-free survival (PFS) and overall survival (OS) by Dmax groups. Shown are the Kaplan-Meier survival curves stratified according to the Dmax groups. The upper part of the figure displays a division into the 3 groups for PFS (**A**) and OS (**B**). Dmax low patients are marked in orange, intermediate patients in gray and high patients in blue. In the lower part, low and intermediate patients are pooled (orange graph) and their survival is compared with that of the high group (blue graph), in each case for PFS (**C**) and OS (**D**)

Survival analyses of Dmax-based groups were plotted as Kaplan Meier curves in Fig. 6. Stratification by Dmax group showed minor differences between the survival curves for PFS (Fig. 6A) that was not significant (*p* = 0.093). In addition, we detected differences in median PFS, which was 369 days in Dmax low group, 94 days in the intermediate group and 91 days in the high group. Dmax high patients display a shorter median OS with 333 days compared with was 632 days for Dmax low and 657 days for Dmax intermediate patients. Regarding OS, no significant separation of the survival curves was observed (Fig. 6B; *p* = 0.348). When the Dmax low and high groups were combined, they showed a significantly (*p* = 0.030) better PFS than the high group (Fig. 6C), but no significant differences (*p* = 0.151) in OS (Fig. 6D) were observed.

In the Dmax bulk-based groups median PFS was 369 days in low, 90 days in intermediate and 97 days in the high patients. Median OS was 632 days in low, 610 days in Dmax bulk intermediate and 404 days in high group. In addition, there were no significant differences between the course of the survival curves of PFS and OS for the stratification according to Dmax bulk groups (Supplementary Fig. 2; all *p* > 0.05).

## Discussion

In our study of r/r NHL patients treated with CAR T-cells in later line setting, patients with a high Dmax had a shorter PFS but no significant differences in OS. Median tumor burden was also significantly higher in the Dmax intermediate and high groups compared to the low group, but patients depth of response was independent of tumor dissemination. Dmaxbulk did not improve risk stratification by tertiles but was significantly associated with PFS and OS in Cox regression. Future studies in lymphoma patients undergoing CART should prospectively assess the value of Dmax as a potential new imaging biomarker for predicting response and survival.

A recent comparison of 19 studies investigating Dmax in conventionally treated lymphomas showed promising results for risk stratification and survival prediction, although the studies were quite heterogeneous [22]. Some publications suggested that combining Dmax with other metabolic biomarkers, such as MTV and interim PET-based response, may better stratify the risk of relapse or death. Some groups proposed that a combination of Dmax and MTV helped to improve stratification of LBCL patients [23, 24]. Others see a benefit by adding a combination of several imaging features including MTV [25]. The literature on radiomics in CAR T-cell therapy is very scarce, but some studies show that the combination of several PET-based radiomic features outperform conventional PET parameters in terms of prognosis estimation [26, 27]. MTV is also associated with survival in context of CART [28, 29]. However, further validation in larger prospective cohorts is needed to determine the best combination of Dmax with semi-quantitative PET parameters and/ or radiomic features in the context of CART.

In addition, the type of the lymphoma also seems to play a role. A study that examined MCL patients showed no significant influence of Dmax on the prognosis [30]. The study landscape in HL is heterogeneous with studies that demonstrated a prognostic value of Dmax, but also other studies in which this effect was not seen. The studies in patients with LBCL almost consistently showed a possibility of risk stratification for PFS and OS by Dmax. Our study included mainly LBCL patients, which would be consistent with the significant differences in PFS. On the other hand 12.9% also had a MCL that may affect the prognostic value of Dmax regarding OS. Regardless of lymphoma type and Dmax, our results indicate a high efficacy of CART, as all patients were able to achieve a good depth of response regardless of their baseline Dmax. In addition, there were no significant differences in the DS of the 90 day PET/CT and all patients were also able to achieve a good metabolic response independent of tumor dissemination.

Mirshahvalad et al. demonstrated that in the context of CAR-T cell therapy, Dmax was prognostic for PFS but not for OS.^20^ This aligns with our findings, although their reported cut-off value of 14 cm was considerably lower than in our study. In their analysis, a Dmax of 60 cm on post-treatment scans emerged as an adverse prognostic factor. In contrast, Girum et al. specifically showed that a baseline Dmax of 60 cm in patients with DLBCL undergoing conventional therapy predicted both shorter PFS and OS.^24^ This threshold is close to the cut-off of 57.8 cm identified in our study. Furthermore, consistent with several other studies, the inclusion of surrogate markers of tumor burden (such as LDH, SPD, and MTV) enhanced the prognostic value of Dmax.

Dmax reflects the spatial dissemination of disease and therefore primarily predicts early relapse, making its association stronger with PFS than with OS. In contrast, OS is influenced by post-progression therapies, heterogeneity in subsequent management, and competing risks, which may dilute the prognostic impact of baseline Dmax. Limited follow-up and fewer OS events may further reduce statistical power. Consistent with our findings, Mirshahvalad et al. also reported an association of Dmax with PFS but not with OS at baseline [20]. 

One possible limitation that underlies the assessment of Dmax is the lack of standardized measurement procedures. While most studies show very good reproducibility, one study showed only moderate reproducibility in Dmax measurement, especially between a fully automated software and physicians [31–33]. Moreover, most of the current studies investigated the effects of Dmax in patients with newly diagnosed lymphoma. In contrast, lymphoma patients treated with CART often suffer from refractory or relapsed disease with further disseminated lymphoma lesions. In these cases, the measurement of Dmax is particularly challenging, especially in the absence of a PET component. In the recently published review, the median Dmax was in the range of 20 to 66 cm.^22^ The median Dmax in our study was 40 cm, which is fairly in the middle of the literature values. The Dmax low group showed median values below the limit at 11 cm and the Dmax high group above the limit at 70 cm. Taken together, the Dmax values in the CAR were comparable to those of lymphomas treated with first-line therapy.

Another challenge, at least in our study, is the precise classification and characterization of the Dmax intermediate group. This is a very heterogeneous group with a higher tumor burden and LDH than the Dmax high group, but also the longest median PFS and OS of all groups. In terms of depth of response, this group includes the patient with the worst DoR, but also many patients with a very good DoR. Bridging therapy could also lead to a shift between the groups. In most cases, patients with poorer tumor dynamics and a higher tumor burden receive bridging therapy. Since in our study Dmax was measured at the time of lymphodepletion, i.e. after a potential bridging therapy, some patients with no longer metabolically active disease could have fallen from the Dmax high to the intermediate group. When looking at Table 1, it is noticeable that the Dmax intermediate patients have the highest rate of bridging therapies, even slightly higher than the Dmax high group. This could also partly explain the higher morphologically measured SPD and the increased LDH in the Dmax intermediate group.

To our knowledge, only limited literature is available on the prognostic impact of Dmax and Dmax_bulk_ in the context of NHL treated with CAR T-cell therapy. Our study has limitations that must be taken into account. First, it is a single center study with a limited number of subjects. This may limit the interpretation of the association of Dmax and Dmax bulk with survival. Second, some patients had to be excluded because there was no measurable disease on CT, which is also a limitation of imaging prognostic indices in routine clinical practice. Third, some patients had only CT without PET at the time of 90-day FU, so there may be a possible redistribution of Deauville scores among Dmax-based groups.

To conclude, baseline Dmax before lymphodepletion of CART treated lymphoma patients showed prognostic value for PFS stratification. In the context of CART in later line lymphoma treatment, Dmax is readily available at baseline imaging and holds potential as a novel prognostic imaging biomarker. Future research should prospectively assess the value of Dmax in larger patient samples and whether the combination of Dmax with other imaging biomarkers as MTV or radiomics has possible benefits in terms of outcome and survival prediction in lymphoma patients undergoing CART.

## Electronic Supplementary Material

Below is the link to the electronic supplementary material.

Supplementary Material 1

## Data Availability

The datasets generated during and/or analysed during the current study are available from the corresponding author on reasonable request.

## Abbreviations

BL: Baseline
CART: Chimeric antigen receptor T-cell therapy
CR: Complete response
DoR: Depth of response
DS: Deauville score
FDG: Fluorodeoxyglucose
FU: Follow-up
IPI: International prognostic index
IQR: Interquartile range
LBCL: Large B-cell lymphoma
MCL: Mantle-cell lymphoma
NHL: Non-Hodgkin lymphoma
ORR: Overall response rate
OS: Overall survival
PET/CT: Positron emission tomography-computed tomography
PD: Progressive disease
PFS: Progression-free survival
PR: Partial response
r/r: Relapsed or refractory
SD: Stable disease
SPD: Sum of product diameters
TB: Tumor burden
TL: Target lesion
