# Supplementary material for: Predictive value of maximum tumor dissemination (Dmax) in lymphoma patients treated with CD19-specific CAR T-Cells
Source: Cancer Imaging. 2025 Nov 22;25:135. doi: 10.1186/s40644-025-00959-w (PMC12670812; doi:10.1186/s40644-025-00959-w)
Supplement: Supplementary file 1 — Supplementary Material 1 [file 40644_2025_959_MOESM1_ESM.docx]

SUPPLEMENTS

| **Covariate** | **HR (PFS)** | **p-value (PFS)** | **HR (OS)** | **p-value (OS)** |
| --- | --- | --- | --- | --- |
| Dmax absolute | 1.006 | 0.171 | 1.004 | 0.351 |
| Dmax > 57.8 cm | **1.821** | **0.021** | 1.581 | 0.104 |
| Dmaxbulk absolute | 1.007 | 0.153 | 1.006 | 0.294 |
| Dmaxbulk > 55.2 cm | **2.429** | **0.003** | **2.053** | **0.022** |
| ECOG (Ref. 0)   - 1 - 2 - 3 | 1.637  2.989  3.088 | 0.173  **0.011**  **0.030** | **2.396**  **2.791**  **4.903** | **0.041**  **0.038**  **0.005** |
| IPI (Ref. 0)   - 1 - 2 - 3 - 4 - 5 | 1.000  1.173  2.076  **3.278**  **3.032** | 0.999  0.705  0.066  **0.005**  **0.033** | 1.000  0.910  1.474  **2.346**  1.623 | 0.999  0.836  0.365  **0.050**  0.397 |
| SPD absolute | 1.000 | 0.286 | 1.000 | 0.101 |
| SPD > 8,503.7 mm^2^ | 1.633 | 0.109 | **2.002** | **0.050** |
| LDH absolute | 1.000 | 0.064 | **1.000** | **0.039** |
| LDH elevated | **2.731** | **< 0.001** | **2.069** | **0.016** |
| Ann Arbor Stage (Ref. Stage I)   - II - III - IV | 1.388  1.905  **3.138** | 0.514  0.222  **0.010** | 1.806  1.785  2.317 | 0.270  0.310  0.085 |

**Suppl. Table 1:** Univariate Cox regression.

| **Covariates** | **HR (PFS)** | **p-value (PFS)** | **HR (OS)** | **p-value (OS)** |
| --- | --- | --- | --- | --- |
| Dmax > 57.8 cm  + Dmaxbulk > 55.2 cm | 1.266  2.011 | 0.511  0.088 | 1.103  1.898 | 0.807  0.151 |
| Dmax > 57.8 cm  + SPD > 8,503.7 mm^2^ | **2.175**  1.445 | **0.030**  0.148 | 1.510  1.930 | 0.145  0.065 |
| Dmax > 57.8 cm  + LDH elevated | **1.786**  2.703 | **0.026**  **< 0.001** | 1.518  **2.020** | 0.139  **0.020** |
| Dmax > 57.8 cm  + ECOG   - 1 - 2 - 3 | 1.579  1.685  **2.577**  **2.630** | 0.103  0.151  **0.032**  **0.067** | 1.380  **2.423**  2.457  **4.310** | 0.295  **0.038**  0.078  **0.011** |
| Dmax > 57.8 cm  + LDH elevated  + ECOG   - 1 - 2 - 3 | 1.659  **2.509**  1.509  1.793  2.295 | 0.067  **0.001**  0.260  0.198  0.113 | 1.406  **1.978**  2.310  1.889  **4.220** | 0.258  **0.028**  0.052  0.222  **0.012** |
| Dmaxbulk > 55.2 cm  + SPD > 8,503.7 mm^2^ | **2.443**  **1.648** | **0.003**  **0.102** | **2.077**  **2.029** | **0.020**  **0.047** |
| Dmaxbulk > 55.2 cm  + LDH elevated | **2.993**  **3.104** | **< 0.001**  **< 0.001** | **2.067**  **2.083** | **0.021**  **0.016** |
| Dmaxbulk > 55.2 cm  + ECOG   - 1 - 2 - 3 | **2.129**  1.663  **2.620**  2.597 | **0.015**  0.161  **0.028**  0.070 | 1.716  **2.331**  2.380  **4.064** | 0.104  **0.047**  0.087  **0.015** |
| Dmaxbulk > 55.2 cm  + LDH elevated  + ECOG   - 1 - 2 - 3 | **2.692**  **2.811**  1.429  1.755  1.906 | **0.002**  **< 0.001**  0.330  0.211  0.230 | **1.769**  **1.989**  2.187  1.839  **3.832** | 0.083  **0.027**  0.070  0.238  **0.021** |

**Suppl. Table 2:** Multivariate Cox Regression.

**Supplementary Fig. 1:** Incidence of CRS and ICANS according to Dmax and Dmax bulk groups. Dmax and Dmax bulk intermediate and high group were significantly associated with increased rates of both CRS and ICANS (p-values indicated).

**Supplementary Fig. 2**: **Survival analyses of progression-free survival (PFS) and overall survival (OS) by Dmax bulk groups.** Depicted are the survival curves stratified according to the Dmax bulk groups. The upper panel displays a division into the 3 groups for PFS (A) and OS (B). Dmax bulk low patients are marked in orange, intermediate patients in gray and high patients in blue. In the lower part, Dmax bulk low and intermediate patients are pooled (orange graph) and their survival is compared with that of the high group (blue graph), in each case for PFS (C) and OS (D).
